# Ultra-Precision Cutting and Characterization of Reflective Convex Spherical Blazed Grating Elements

**DOI:** 10.3390/mi13071115

**Published:** 2022-07-15

**Authors:** Huang Li, Xiaoqiang Peng, Chaoliang Guan, Hao Hu

**Affiliations:** 1 College of Intelligence Science, National University of Defense Technology, Changsha 410073, China; li_huang6002@163.com (H.L.); chlguan@nudt.edu.cn (C.G.); tiny_hh@139.com (H.H.); 2 Laboratory of Science and Technology on Integrated Logistics Support, National University of Defense Technology, Changsha 410073, China; 3 Hu’nan Key Laboratory of Ultra-Precision Machining Technology, Changsha 410073, China

**Keywords:** ultraprecision, blazed gratings, convex spherical substrate, Poisson burr, machining accuracy, grating layouts

## Abstract

In this work, based on the diffraction principle of reflective blazed grating, the structure size of the convex spherical blazed grating unit is determined, the machining accuracy of the convex spherical blazed grating is formulated, the effects of tool nose radius and Poisson burr on the diffraction efficiency of the convex spherical blazed grating are analyzed, and the performances of cutting convex gratings with microcrystalline aluminum RSA6061 and RSA6061+ chemically plated NiP for two workpiece materials are compared. A convex spherical blazed grating with a radius of curvature R = 41.104 mm, substrate diameter 14 mm, grating density 53.97 line/mm, and blaze angle of roughly 3.8° is turned by a four-axis ultra-precision machining system by adjustment of the cutting tool, workpiece material, and cutting parameters, as well as modification of the layouts of the blazed grating on the convex sphere. The results of the testing of convex spherical blazed grating elements in both layouts show that the size error of the grating period is close for both layouts, the size error of grating height is smaller in the equal-along-arc layout, the blaze angle error in the equal-along-projection layout is only 0.74%, and the average roughness of the blazed surface is less than 5 nm to meet the processing quality requirements of the reflective convex spherical blazed grating. The greater the blaze angle accuracy of the blazed grating, the higher its diffraction efficiency, so the grating element with an equal-along-projection layout has a higher diffraction efficiency than the grating element with an equal-along-arc layout. RSA6061+ chemically plated NiP material is superior to RSA6061 material in Poisson burr height and blazed surface roughness, which is more suitable for Offner-type imaging spectrometers in the spectral range 0.95–2.5 μm (SWIR).

## 1. Introduction

Microstructured functional surfaces are widely used in advanced science and industry for their excellent properties, but their performance is limited by the surface quality of the fabrication method [1]. Examples of microstructured optic elements are Fresnel lenses and diffractive optical elements (DOEs), which can be used to improve optical properties, such as beam shaping or correction of aberrations, while reducing the weight of optical systems. Diffraction gratings [2,3,4] are optical elements with a periodic, subwavelength-scale range of optical functional structures that can spatially modulate the amplitude or phase of the incident light, and are widely used in spectral instruments, laser modulation, optical metrology, information processing, thin-film optics, polarization optics, and many other fields.

Compared with other spectroscopy systems (planar grating spectroscopy, Fourier transform spectroscopy, filter spectroscopy, liquid crystal tunable filter, acousto-optical modulation, etc.), the imaging spectroscopy instrument based on convex grating spectroscopy has the advantages of large optical relative aperture, good linearity of dispersion, compact structure, and good image imaging quality. The Offner imaging spectrometer [5] applies a classical grating that exhibits good field correction performance. However, most of the available fabrication techniques, such as direct ruling, holography, photolithography, or electron beam writing, are typically applied to simple-shaped grating surfaces, such as planar substrates or spherical substrates with low steepness. Advanced Machine and Optical Systems (AMOS), Belgium, demonstrated the feasibility of ultra-precision machining of free-form gratings (FFG), i.e., blazed gratings can be fabricated on surfaces without any rotational symmetry using a cost-effective single-point diamond turning (SPDT) method [6].

With the improvement of ultra-precision machining technology, the preparation of surface optical functional microstructures has become a hot research topic in the field of precision machining. Traditional mechanical ruling is no longer suitable for the precision fabrication of convex blazed gratings, and the single-point diamond turning process has been proven to be suitable for processing advanced optical functional surfaces, such as discontinuous microstructures and free-form surfaces, due to its high machining accuracy and determinism [7,8]. Ultra-precision machining of free-form blazed gratings can be a reliable alternative to electron beam lithography in holographic manufacturing [9,10]. By synchronous linkage of multiple axes of ultra-precision machine tools and precise control of the tool–workpiece relative position according to the geometry of the convex grating, grating structures with variable pitch can be fabricated. Di Xu et al. [11] used a Moore Nanotechnology 350 FG ultra-precision five-axis machine to cut convex gratings of variable pitch on brass C46400 substrate with a grating density of 300 lines/mm and blazed surface roughness of about 10–15 nm RMS and 30–45 nm Rz. The design was suitable for the 500–1100 nm spectral range. ChaBum Lee et al. [12] used the diamond tool interferometric ruling method to prepare blazed gratings on nickel-plated molds with blazed grating dimensions: a grating period of 2.0 μm, grating height of 0.2 μm, and blaze angle of 5.86°, and the diffraction efficiency was 87.6% after replication. Chun-Wei Liu et al. [13] studied the effects of shape design, grating period, and cutting speed on the processing performance of the mold using diamond turning on a brass roller, and the optical measurement results showed that the performance of the subwavelength grating matched the design at different incidence angles, so the high-precision mold by diamond turning is a feasible way to ensure the continuous mass production of subwavelength gratings. Tan N Y J et al. [14] developed the Continuous Rotating Freeform Shaping (CRFS) algorithm and combined it with the Slow Slide Servo system to machine high-curvature radial gratings and free-form gratings using a five-axis ultra-precision machine with an average grating period of 12.5075 μm; the average blaze angle was 8.25 ± 0.01°, and the surface roughness Sa was 0.015 μm and 0.018 μm, respectively, with a profile deviation of less than 0.3%. De Clercq C [6] used a five-axis ultra-precision lathe and a single-point diamond tool to machine free-form gratings with a diameter of 35 mm and a radius of curvature of 80 mm on a workpiece chemically coated with NiP on the surface of an aluminum 6061T6 substrate, and showed that the roughness measured in a single groove was close to 4 nm RMS and the blaze angle was 1.82°. Based on the five-axis ultra-precision single-point diamond lathe cutting process, Zheng Zhizhong et al. [15] calculated the diamond tool tip movement error, the standard deviation range of movement interval, and the processing deviation of grating inscription position, and analyzed the effect of tool nose wear on the grating diffraction efficiency, successfully developing a convex blazed grating with a substrate of 6061 aluminum, a curvature radius of 70 mm, inscription density of 60 line/mm, and diameter of 52 mm, whose maximum relative diffraction efficiency was greater than 80% and average relative diffraction efficiency was greater than 60% in the spectral range of 1000~2500 nm. Graham C et al. [16] designed an all-aluminum, rugged, lightweight, free-form grating-based near-infrared hyperspectral moisture sensing imager, FYMOS, which was machined on a five-axis ultra-precision diamond machine. Bourgenot C et al. [17] from Durham University, UK, reported the technical challenges and progress in preparing such curved blazed gratings on an ultra-precision five-axis Moore machine, describing their application in an integrated grating imaging spectrometer (IGIS) integral field unit prototype, in which the free-form gratings were used as optical pupil reflectors. Bourgenot C et al. [18] also discussed new opportunities for free-form gratings machined with diamond, and identified NiP as the substrate providing the best roughness and profile quality in a comparison of free-form gratings machined with RSA aluminum 6061 and RSA aluminum 443 surfaces chemically coated with NiP with an average roughness of 2.5 nm RMS per blazed surface. The integrated design of structure and function is the development trend of future imaging spectroscopy instrument design, and the use of the same metal can effectively avoid the influence of the bimetallic effect on the system stability. All of the preceding studies used five-axis ultra-precision machines to manufacture convex blazed gratings, but did not investigate the effect of Poisson burrs on the top of the grating on its diffraction efficiency, nor did they investigate the relationship between grating layout on the convex substrate and machining accuracy.

**Figure 1:**
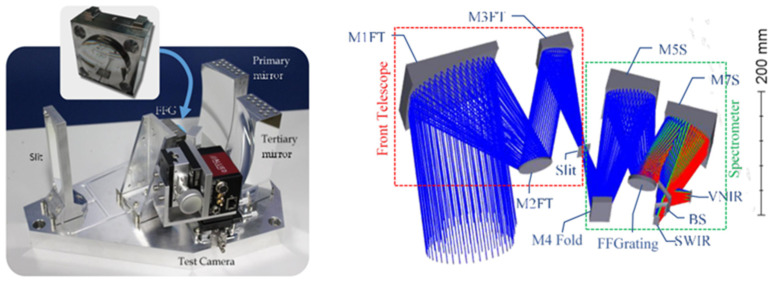
ELOIS spectroscopy instruments. “Reprinted/adapted with permission from Ref. [19]. 2019, Moreau V”.

The use of homogeneous metals can effectively avoid the effect of bimetallic effects on system stability, which is the trend of future imaging spectroscopy instrument design. Moreau V et al. [19] introduced ELOIS (as shown in Figure 1) and CHIMA, two innovative all-aluminum spectroscopy instruments based on free-form straight-grained diffraction gratings that provide approximately four times smaller solutions than Offner-Chrisp spectrometers with comparable performance. As an alternative material for grating elements, microcrystalline aluminum RSA6061, a new ultra-fine grain aluminum alloy with good cutting properties can be used.

Based on the imaging spectral range and diffraction requirements of an imaging spectrometer, this paper determines the structure size of a convex spherical blazed grating unit based on the diffraction principle of reflective blazed grating, develops the machining accuracy of convex spherical blazed grating, analyzes the influence of tool nose radius and Poisson burr on the diffraction efficiency of convex spherical blazed grating, investigates the effect of the layout of blazed grating on the convex surface on the machining accuracy, compares the performance of different workpiece materials cutting convex grating, and obtains high-precision reflective convex spherical blazed grating element.

## 2. Reflective Blazed Grating Technical Requirements

### 2.1. Diffraction Principle of Blazed Grating

**Figure 2:**
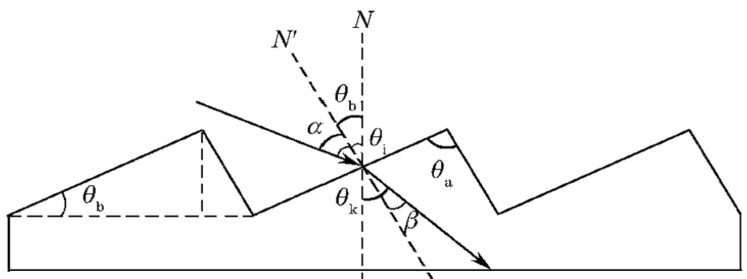
Structure model of reflective blazed grating.

Wood [20] invented the “blazed” grating approach in 1910 to improve grating diffraction efficiency, which involves modifying the energy distribution of light at each diffraction level by changing the geometry of the grating groove. If the grooved surface of the grating is not parallel to the normal of the grating, i.e., there is a small angle *θ*_b_ between the two (blaze angle, as shown in Figure 2), the grating can transfer the energy of the zero-order spectrum to the desired-order spectrum to achieve the “blaze” of the order, which is called blazed grating. The wavelength corresponding to the maximum light intensity is called the blaze wavelength. The design of the blaze angle allows the grating to be applied to a particular order of the spectrum in a particular waveband. In Figure 2, N is the grating normal, N’ is the normal of the grating groove (i.e., the normal of the blazed surface), *θ*_i_ is the incidence angle, and *θ*_k_ is the diffraction angle.

According to the grating equation.

(1) $$d\left(\sin\theta_{i}+\sin\theta_{k}\right)=k\lambda$$

Choosing a suitable blaze wavelength *λ_b_*, the blaze angle *θ_b_* can be deduced from Equation (1):

(2) $$\theta_{b}=\arcsin\frac{k\lambda_{b}}{2d}$$

where *k* is the diffraction order; *d* is the grating period.

From Equation (1) to Equation (2), the blaze angle *θ_b_* is related to the grating.

(3) $$\theta_{b}=\arcsin(\frac{\sin\theta_{i}\pm \sin\theta_{k}}{2})$$

When the incident light is parallel to the line AB, the grating top angle *θ_a_* is obtained as follows:

(4) $$\theta_{a}=90^{\circ }-\theta_{b}+\left|\theta_{i}\right|$$

where *θ_b_* is the blaze angle, °; *θ_i_* is the incidence angle of the light.

The grating period *d* satisfies the following equation:

(5) $$d=\frac{1000}{f}$$

where *d* is the grating period, μm; *f* is the grating frequency (density), line/mm.

In the grating unit structure △OAB, it is known from the sine theorem:

(6) $$\frac{d}{\sin\theta_{a}}=\frac{OA}{\sin(180^{\circ }-\theta_{a}-\theta_{b})}$$

where *θ_a_* is the top angle of the grating; *OA* is the length of the blazed surface, μm.

The grating height (groove depth) *h* can be given by the following equation.

(7) $$h=OA\sin\theta_{b}$$

Therefore, from Equation (5) to Equation (7), it can be shown as follows.

(8) $$h=\frac{1000\sin(180^{\circ }-\theta_{a}-\theta_{b})}{f\sin\theta_{a}}\sin\theta_{b}$$

Therefore, the grating height (groove depth) *h* is determined by the grating top angle, blaze angle, and grating density.

### 2.2. Accuracy Requirements of Convex Spherical Blazed Grating

#### 2.2.1. Dimensional and Surface Accuracy

**Figure 3:**
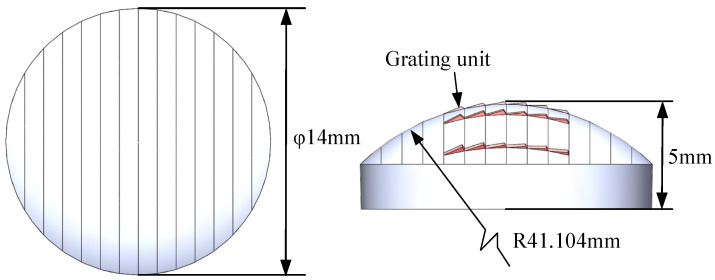
Workpiece size of convex spherical blazed grating.

The specifications of the convex spherical blazed grating, which was designed by Shanghai Institute of Technical Physics, Chinese Academy of Sciences, are shown in Table 1. The designed application spectral range is 0.95–2.5 μm (SWIR), and the corresponding diffraction angle is 17.629° when the incidence angle is −25.962°. According to the specifications of convex spherical blazed grating and the Equations (1)–(8), the geometry of convex spherical grating can be calculated as follows: curvature radius R = 41.104 mm, base diameter of 14 mm, grating blaze angle *θ_b_* = 3.8681868°, grating top angle *θ_a_* = 112.0938132°. From the grating density of 53.97 line/mm, the grating period can be calculated as *d* = 18.5288123 μm and the grating height *h* = 1.212900274 μm. The dimensions of the convex spherical blazed grating are shown in Figure 3. Since various errors in convex spherical blazed gratings can directly affect the grating diffraction efficiency, rigorous demands are placed on the processing accuracy of the diffraction element. For optical components, the roughness of the grating blaze surface needs to be less than 10 nm to meet the optical quality requirements [21]. The dimensional error of the grating period is required to be between ±3 μm, the dimensional deviation is less than 3%, and the blaze angle error is required to be between ±0.05°. To achieve such high machining quality requirements, machining by forming method can effectively replicate the shape of the tool and ensure the dimensional accuracy of the finished product.

#### 2.2.2. Effect of Shape Accuracy on Diffraction Efficiency

In cases where the machine tool itself meets the accuracy requirements, it is still necessary to consider the stability of the environment and the machine error caused by thermal control and tool wear during a long cycle time. The long cycle time of the machining process is not enough to keep the forming error within the allowable range; the forming error needs to be solved by temperature control and long-term drift experiment. The single-point diamond tool tip wears during the cutting process, which can lead to residual rounding at the bottom of the grating, resulting in an invalid diffraction area. As the tip wears, the tool nose radius increases accordingly, the residual rounding angle at the bottom of the grating increases with it, and the grating diffraction efficiency decreases instead [15]. At the same time, Poisson burrs inevitably remain at the top of the blazed grating due to the plastic flow of the material [22]. When the flow stress equals the shear yield strength, the plastic workpiece begins to have plastic side flow. Under the action of high pressure, the workpiece material flows in the direction of least resistance. If the pressure is greater than the shear yield strength, the workpiece material will flow out of the free surface at the front end, forming Poisson burrs. The relationship between Poisson burr height *h_p_* and undeformed chip thickness *d_c_* can be shown as follows [23]:

(9) $$h_{p}=(k_{1}\ln\frac{E\cot\frac{\beta }{2}}{\sigma }+k_{2})d_{c}-d_{c}$$

where, *h_p_* is Poisson burr height, μm; *d_c_* is undeformed chip thickness, μm; the parameters *k*_1_ and *k*_2_ can be calibrated by ultra-precision cutting experiments [24]; *E* is the material elastic modulus, GPa; *σ* is the flow stress, Mpa; *β* is the included angle of the tool.

**Figure 4:**
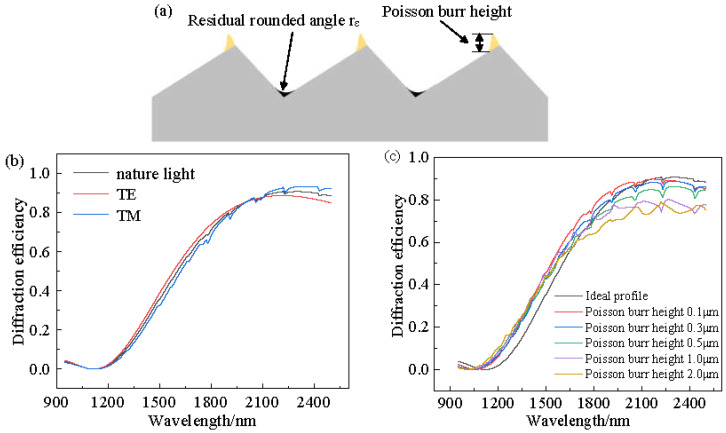
Simulation of the diffraction efficiency of the convex spherical blazed grating. (**a**) Residual rounded angle and Poisson burr. (**b**) Diffraction efficiency of ideal profile grating. (**c**) Grating diffraction efficiency under the influence of different Poisson burr height.

The distribution locations of the residual rounding angle and Poisson burrs are shown in Figure 4a. Both of these have an effect on the grating diffraction efficiency. The diffraction efficiencies of natural light, TE polarized light, and TM polarized light under ideal contours of the convex spherical blazed grating were simulated using PCGrate software, which uses the exact boundary integral equation method. The simulation parameters were consistent with the structural design parameters, and the simulation results are shown in Figure 4b. The variation of diffraction efficiency in the spectral range of 0.95–2.5 μm when the Poisson burr height values are 0 μm, 0.1 μm, 0.3 μm, 0.5 μm, 1.0 μm, and 2.0 μm for the actual profile of the convex blazed grating. From Figure 4c, it can be seen that with the increase in Poisson burr height, the grating diffraction efficiency in the spectral range gradually decreased. When the Poisson burr height was less than 0.5 μm, the effect of the Poisson burr on the grating diffraction efficiency could be neglected. Therefore, it was necessary to suppress the Poisson burr at the top of the blazed grating and reduce the residual rounding r_ε_ at the bottom of the blazed grating to achieve higher diffraction efficiency.

## 3. Cutting Experiments for Convex Spherical Blazed Grating

### 3.1. Machining Equipment and Tools

**Figure 5:**
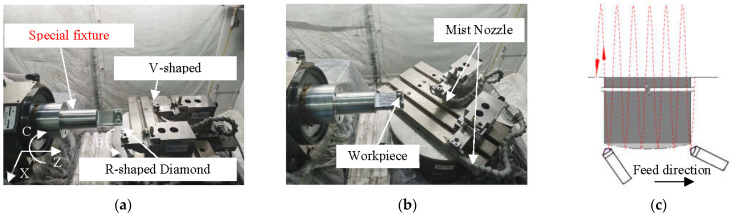
Workpiece and tool clamping methods. (**a**) When machining convex spherical surfaces. (**b**) When machining convex spherical gratings. (**c**) Feed direction of tool.

In order to avoid the machining error caused by the variation of cutting depth, the shaping method of turning was used in the four-axis (XZBC) ultra-precision machining system. As shown in Figure 5a, the special fixture was designed to be mounted on the spindle vacuum chuck. The workpiece was mounted on the side of the special fixture. At this time, the structure of the workpiece and the special fixture after assembly was not rotationally symmetric, so it was necessary to rely on the balance screw on the special fixture for spindle dynamic balancing adjustment before the cutting experiment. This reduced noise effects due to vibrations caused by such loadings, which may be reflected on the surface of the workpiece. The balance deviation was steadily controlled within 2 nm along the axis of the workpiece when the spindle speed was 1000 rpm. The rotating tool holders of the B-axis were mounted side by side with a single-point diamond tool with a large and a small tool nose radius. The V-shaped diamond tool for cutting the blazed grating should be mounted at the center of rotation of the B-axis as far as possible, using the virtual center of the four-axis ultra-precision machine tool for machining. The R-shaped diamond tool with a large tool nose radius was first used to turn the convex spherical substrate so that the curvature center of the convex spherical substrate fell on the spindle centerline. After obtaining a high-quality convex spherical substrate, the tool holder was rotated around the B-axis by the first angle value B1 (calculated by the MATLAB program), and the V-shaped diamond tool nose for machining the blazed grating was aligned with the convex spherical surface. The main cutting motion was the direction of the workpiece rotating with the C-axis. Through stepping motion by X-axis, the cutting depth direction movement of each blazed grating could be achieved. The tool tip moved along the convex spherical generatrix and stepped along the Z-axis direction while deflecting the tool angle through the B-axis to adapt to the change in the blaze angle directly on the convex spherical surface, so as to realize the machining of all radial blazed gratings on the convex spherical substrate surface, as shown in Figure 5b.

In order to improve the forming accuracy of the grating structure, single-point diamond was chosen as the tool material and the geometric parameters of the tool was checked by an optical microscope. For ultra-precision turning by forming method, the included angle of the forming tool was the same as the angle between the grooves of the blazed grating (i.e., the top angle of the blazed grating), and the tool rake angle of 0° was selected to reduce the influence of the tool forming error on the turning accuracy by forming method. In microfabrication, the cutting-edge radius had a great influence on the machining quality [25]. Wu [26] showed that the burr height increased with the increase in the cutting-edge radius. Considering the strength and wear rate of the tool, the cutting-edge radius of the diamond tool was selected as 0.1 μm, and the accuracy of the forming surface was improved by ultra-precision grinding of the cutting edge of the diamond tool. The increase in the radius of the tool nose will increase the area of the invalid diffraction area at the bottom of the blazed grating, thus directly affecting the diffraction efficiency of the convex spherical blazed grating. Thus, considering the wear rate of the diamond tool tip, the tool nose radius was chosen to be 0.1 μm. The geometric parameters of the forming turning tool are shown in Table 2.

The workpiece materials used in this paper were microcrystalline aluminum RSA6061 and RSA6061+ chemically plated NiP. The composition of microcrystalline aluminum RSA6061 mainly consisted of 98.1% aluminum (Al), 0.3% copper (Cu), 1.1% magnesium (Mg), and 0.5% silicon (Si). RSA6061+ chemically plated NiP is a modified layer of high phosphorus nickel-phosphorus alloy with a thickness of 150 μm, and the contents of Ni and P elements are 87.6% and 12.4%. The physical properties of the two workpiece materials and the diamond tools are shown in Table 3. During the cutting process, spray cooling was used at the diamond tool nose, and the air conditioning cooling system of the machining system was turned on to remove the cutting heat generated during the machining process, so that the cutting environment temperature was kept within ±0.1 °C to minimize the machining error caused by the change in the temperature field.

### 3.2. Machining Path Planning

When the center of curvature of the convex spherical substrate falls on the machine spindle, there are two different layouts of the blazed grating on the convex spherical surface, namely, equal-along-arc and equal-along-projection. The size of the blazed grating unit is the same for both layouts, but the actual cross-sectional profile of the grating is different, resulting in different diffraction efficiency. The two layouts also lead to different coordinates of the tool tip point position, which are calculated as given below.

**Figure 6:**
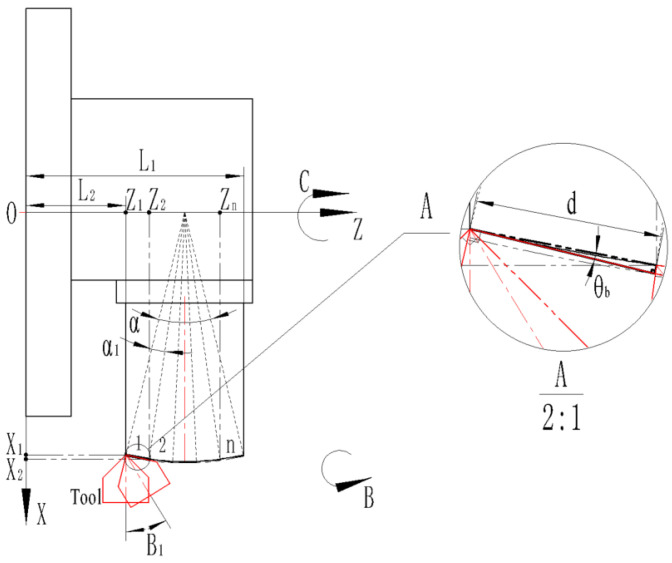
Processing schematic with equal-along-arc layout.

(1) The following Figure 6 is the processing diagram for the convex spherical blazed grating with an equal-along-arc configuration. Each blazed grating corresponds to an equal arc length (i.e., the central angle is equal).

Depending on the relative position of the machine tool and the workpiece, the following geometric relationships are satisfied:

(10) $$L_{1}-L_{2}=2R\sin\frac{\alpha }{2}$$

where *L*_1_ is the distance between the coordinate origin and the farthest end of the side of the workpiece, mm; *L*_2_ is the distance between the coordinate origin and the nearest end of the side of the workpiece, mm; *R* is the substrate curvature radius of the workpiece, mm; *α* is the central angle corresponding to the chord length (*L*_1_ − *L*_2_):

(11) $$d=2R\sin\frac{\alpha_{1}}{2}$$

where *d* is the blazed grating period, μm; *α*_1_ is the central angle corresponding to the chord length *d* (i.e., grating period), °.

Thus, *n* blazed gratings can be arranged on a convex spherical substrate.

(12) $$n=\left[\frac{\alpha }{\alpha_{1}}\right]\;([]\;\mathrm{is}\;\mathrm{the}\;\mathrm{upward}\;\mathrm{rounding}\;\mathrm{function})$$

The *α*-central angle is divided n equal parts, where α_1_ = α_2_ = … = α*_n_*.

The blazed grating closest to the coordinate origin is defined as the 1st blazed grating, followed by the 2nd, 3rd, …, nth blazed grating along the +Z-axis direction in turn.

Therefore, the Z-axis coordinate point *Z_n_* of the nth grating’s blaze angle can be calculated:

(13) $$Z_{n}=L_{1}-\frac{L_{1}-L_{2}}{2}-(R-h)\cos[(n-1)\alpha_{1}+\frac{180^{\circ }-\alpha }{2}]$$

where *h* is the blazed grating height, μm.

Therefore, the X-axis coordinates point *X_n_* of the nth grating’s blaze angle can be calculated.

(14) $$X_{n}=(R-h)\sin[(n-1)\alpha_{1}+\frac{180^{\circ }-\alpha }{2}]$$

Therefore, the B-axis coordinates point *B_n_* of the nth grating’s blaze angle can be calculated:

(15) $$B_{n}=180^{\circ }-[(90^{\circ }-\frac{180^{\circ }-\alpha }{2})+\frac{180^{\circ }-\alpha_{1}}{2}+\theta_{b}+\frac{\beta }{2}]+(n-1)\alpha_{1}$$

where *θ_b_* is the grating blaze angle; *β* is the included angle (in this case the included angle is 110°).

**Figure 7:**
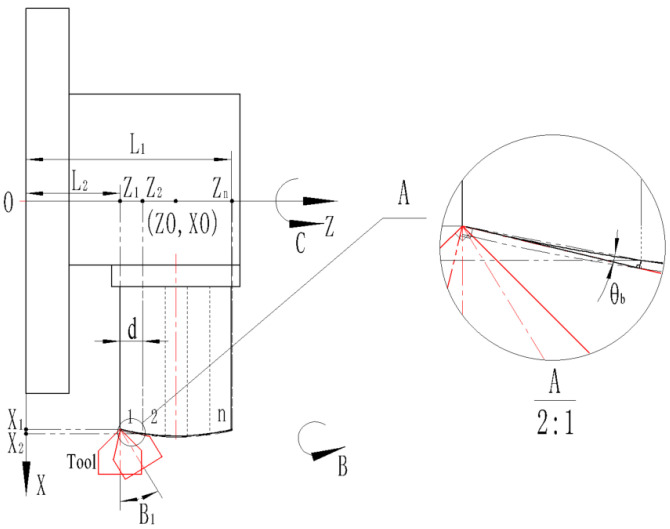
Processing schematic with equal-along-projection.

(2) The following Figure 7 is a processing diagram for a convex spherical blazed grating set in equal-along-projection.

On a convex spherical substrate, *n* blazed gratings can be arranged:

(16) $$n=\left[\frac{L_{1}-L_{2}}{d}\right]\;([]\;\mathrm{is}\;\mathrm{the}\;\mathrm{upward}\;\mathrm{rounding}\;\mathrm{function})$$

where *L*_1_ is the distance between the coordinate origin and the farthest end of the workpiece, mm; *L*_2_ is the distance between the coordinate origin and the nearest end of the workpiece, mm; *d* is the blazed grating period, μm.

Since the substrate curvature radius corresponds to the coordinates of the center of the circle as (*ZO*, *XO*), the standard equation of the circle based on the radius of substrate curvature is shown as follows:

(17) $${\left(z-\mathrm{ZO}\right)}^{2}+{\left(x-XO\right)}^{2}={\left(R-h\right)}^{2}$$

where *R* is the substrate curvature radius of the workpiece, mm; *h* is the blazed grating height, μm.

The blazed grating closest to the coordinate origin is defined as the 1st blazed grating, and the 2nd, 3rd, nth blazed grating along the +Z-axis direction in turn.

Therefore, the Z-axis coordinate *Z_n_* of the nth grating’s blaze angle point can be calculated.

(18) $$Z_{n}=L_{2}+(n-1)d$$

Therefore, the X-axis coordinates *X_n_* of the nth grating’s blaze angle point satisfy the following relationship (*X_n_* values are taken as positive solutions).

(19) $${\left(Z_{n}-\mathrm{ZO}\right)}^{2}+{\left(X_{n}-XO\right)}^{2}={\left(R-h\right)}^{2}$$

Therefore, the B-axis coordinates *B_n_* of the nth grating’s blaze angle point can be calculated:

(20) $$B_{n}=\frac{180^{\circ }-\beta }{2}-\arctan\frac{X_{n}{}_{+1}-X_{n}}{Z_{n+1}-Z_{n}}-\theta_{b}$$

where *θ_b_* is the grating blaze angle; *β* is the included angle (in this case the included angle is 110°).

### 3.3. Cutting Parameter Selection

The processing of convex spherical blazed gratings with equal-along-projection and equal-along-arc is carried out in two steps, with the same cutting parameters for the two different convex spherical grating layouts. Whether processing convex spherical substrate or blazed gratings on a convex spherical substrate, the cutting speed *v_c_* of a diamond tool is defined as the tangential speed between the contact point of the tooltip and the corresponding rotating circle around the spindle, that is, peripheral cutting speed *v_p_*:

(21) $$v_{c}=v_{p}=\frac{2\pi R_{n}n_{c}}{60}$$

where *R_n_* is the radius of gyration at the point of contact between the tool tip and the workpiece, mm; *n_c_* is the spindle speed of the C-axis, rpm.

**Figure 8:**
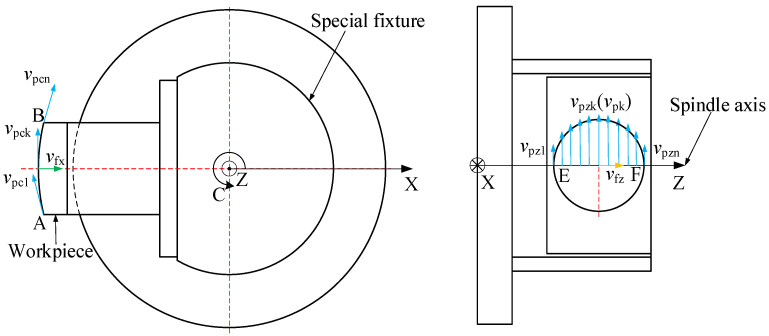
Schematic diagram of peripheral cutting speed distribution along the Z-axis diameter on the workpiece.

For the machining of blazed gratings, the radius *R_n_* (*R_c_*) of gyration around the C-axis at any point on the arc $\overset{⌒}{\mathrm{AB}}$ is equal, so the tangential speed of any point on the arc $\overset{⌒}{\mathrm{AB}}$ is the same. The cutting speed for cutting the same grating groove remains unchanged, that is, *v_pc_*_1_ = *v_pc_*_2_ = …= *v_pck_* = …= *v_pcn_*. However, the radius *R_n_* (*R_z_*) of gyration around the C-axis corresponding to any point on the arc $\overset{⌒}{\mathrm{EF}}$ in the radial direction of the Z-axis is different, expressed as *R_z_*_1_, *R_z_*_2_, …, *R_zk_*, *R_zn_*, respectively. Its numerical value corresponds to X_1_, X_2_,…, X*_k_*, X*_n_*_,_ calculated by Equations (14) and (19). Therefore, from Equations (14), (19), and (21), the peripheral cutting speed of any grating groove on the arc $\overset{⌒}{\mathrm{EF}}$ can be calculated as *v_pz_*_1_, *v_pz_*_2_, …, *v_pzk_*, *v_pzn_*. The feed rates in the X-axis and Z-axis directions are *v_fx_* and *v_fz_*, respectively, as shown in Figure 8.

For equal-along-projection and equal-along-arc layout, the peripheral cutting speed presents a sinusoidal distribution on arc $\overset{⌒}{\mathrm{EF}}$, as shown in Figure 8. The maximum peripheral cutting speed *v_pz_*_(max)_ appears at the highest point of the arc $\overset{⌒}{\mathrm{EF}}$, and the minimum peripheral cutting speed *v_pz_*_(min)_ appears at the lowest point of the arc $\overset{⌒}{\mathrm{EF}}$.

(1) Convex spherical substrate machining

Convex spherical substrate machining is further divided into rough and finish machining. With other cutting parameters unchanged, the main cutting force and burr height gradually increase with the increase in undeformed chip thickness [27]. Considering that the tool nose radius and cutting-edge radius of the diamond tool used are 0.1 μm, and the larger cutting force easily leads to microchipping of the tool, the rough cutting depth was selected as 8 μm to improve the machining efficiency. During finishing stage, the cutting depth was reduced, the surface quality was better, and the burr size was also smaller. However, as the depth of cut further decreased, the cutting process transformed into a plowing process and no longer produced chips, and the surface quality became worse at this time [28]. Thus, the finish depth of cut was 1.4 μm to improve the convex spherical surface shape accuracy. Under the same cutting depth, the smaller the cutting speed, the larger the shear strain ε, and the more likely the plowing phenomenon [29]. Taking into account the stability of the machine tool and cutting efficiency, the spindle speed *n_c_* was chosen to be 1000 rpm, and the unit displacement along the Z-axis was 2 μm. The feed rate in both the X-axis and Z-axis directions was *v_fx_* = *v_fz_* = 50 mm/min. It can be estimated that the maximum peripheral cutting speed *v_pz_*_(max)_ was 4.328 m/s, and the minimum peripheral cutting speed *v_pz_*_(min)_ was 4.265 m/s. The machining time of the blazed grating can be estimated to be about 0.5 h.

(2) Blazed grating machining

In order to avoid surface quality degradation caused by plowing, plowing phenomenon starts to occur at undeformed chip thickness of 1 μm and surface roughness starts to deteriorate according to previous studies [30]. Since the blazed grating height of *h* = 1.212900274 μm, the undeformed chip thickness was the grating height since the blazed grating was processed only by the one-step method. In order to reduce the radial runout at the axis end of the special fixture, the spindle speed *n_c_* was chosen to be 1000 rpm, and the unit displacement along the Z-axis was all grating periods of *d* = 18.5288123 μm. The feed rate in the Z-axis direction was *v_fz_* = 50 mm/min, and the feed rate in the X-axis direction was *v_fx_* =10 mm/min. It can be estimated that the maximum peripheral cutting speed *v_pz_*_(max)_ was 4.304 m/s, and the minimum peripheral cutting speed *v_pz_*_(min)_ was 4.241 m/s, either equal-along-arc layout or equal-along-projection. Combining the substrate diameter and the grating density, the machining time of the blazed grating can be estimated to be about 2.5 h.

## 4. Cutting Quality Characterization for Convex Spherical Blazed Grating

**Figure 9:**
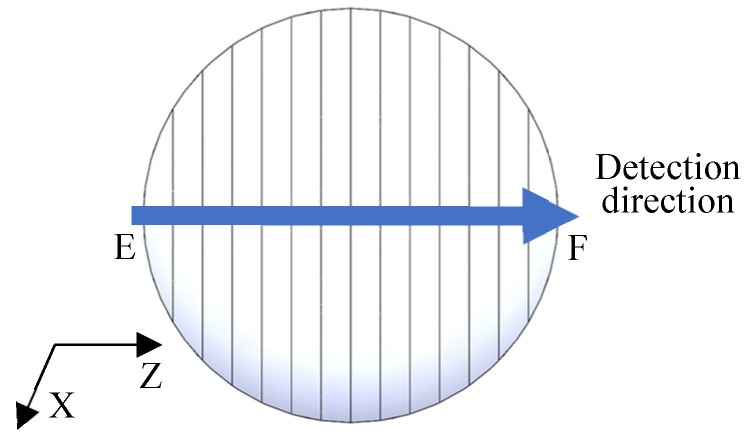
Area of detection for convex grating.

The arithmetic average surface roughness was defined according to ISO25178 standard. There are many different roughness parameters in use, but the arithmetic average surface roughness Ra is by far the most common. Ra is the arithmetic mean of the filtered roughness profiles determined from the deviation of the centerline within the evaluation length. For equal-along-projection, 756 lines need to be machined within the diameter of 14 mm, while for equal-along-arc layout, 760 lines need to be machined within the diameter of 14 mm. Therefore, along the diameter direction of the Z-axis, the surface roughness of the blazed surface was detected every 20 lines, as shown in Figure 9. The Zygo NewView 700 white light interferometer was used to measure the blaze surface roughness Ra of the RSA6061 convex grating and RSA6061+ chemically plated NiP convex grating, as well as the equal-along-arc and equal-along-projection layouts, respectively. At the equal-along-projection layout, a scanning electron microscope was utilized to analyze the surface morphology of RSA6061 convex gratings and RSA6061+ chemically plated NiP convex gratings, as well as comparing the creation of Poisson burrs.

### 4.1. Surface Roughness Distribution in the Diameter Direction

**Figure 10:**
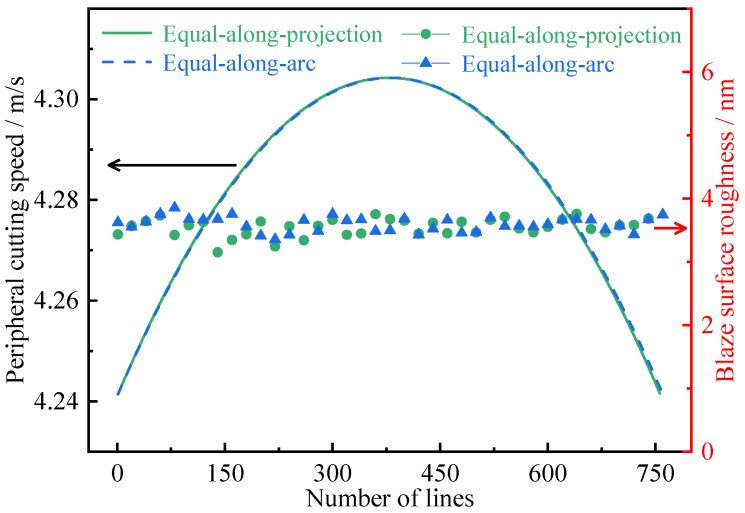
Peripheral cutting speed and surface roughness distribution in the diameter direction.

For equal-along-projection and equal-along-arc layout, peripheral cutting speed and surface roughness distribution in the diameter direction on RSA6061 convex gratings are shown in Figure 10. The difference between the maximum peripheral cutting speed *v_pz_*_(max)_ and the minimum peripheral cutting speed *v_pz_*_(min)_ was only 0.06 m/s. Roughness was distributed 3–4 nm along the Z-axis diameter direction, and was only slightly affected by peripheral cutting speed.

### 4.2. The Effect of Grating Unit Layout on Dimension Accuracy

**Figure 11:**
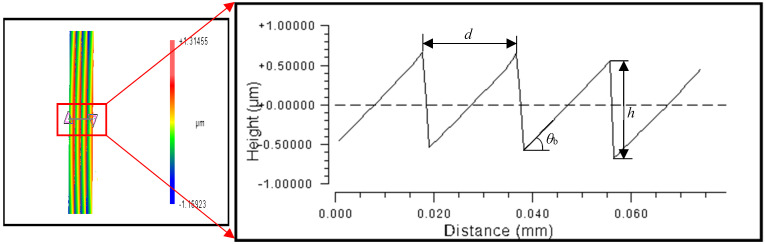
Measurements for grating period, grating height, and blaze angle of blazed grating.

The structural dimensions of the convex blazed grating were measured at equal-along-arc and equal-along-projection layouts using the 20X lens of the Zygo NewView 700 white light interferometer. In the three random sampling zones, the grating period, grating height (groove depth), and blaze angle were all measured. For each of the five grating structures in the three sampling zones, the average value and deviation were computed. The cross-sectional profile of the convex spherical blazed grating is shown in Figure 11. The average values and deviations were calculated for each of the five grating structures in each of the three sampling areas, as shown in Table 4.

**Figure 12:**
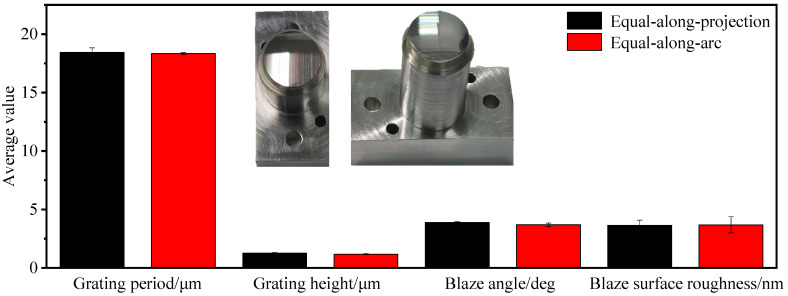
Comparison of grating period, grating height, blaze angle, and blaze surface roughness of blazed grating at different layouts.

Figure 12 shows a physical view of the obtained convex spherical blazed grating elements and a comparison of the values of the grating period, grating height, and blaze angle of the grating unit, where the average value of each dimension is very close, but the dimensional deviation from the design value is different.

**Figure 13:**
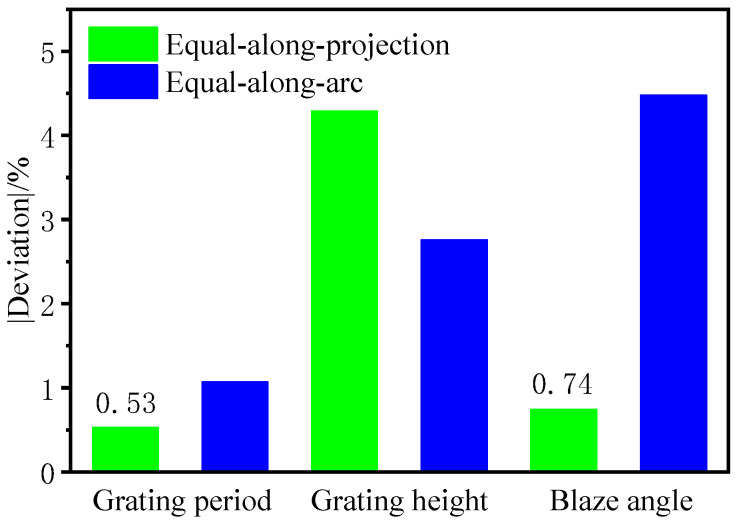
Comparison of dimensional deviations of blazed grating at different layouts.

As shown in Figure 13, comparing the convex spherical blazed grating samples of the two arrangements, it can be concluded that the grating period with an equal-along-projection layout was close to that with an equal-along-arc layout; the deviation of the grating height with equal-along-arc layout was better than that with the equal-along-projection layout; the accuracy of the blaze angle with equal-along-projection layout was better than that with the equal-along-arc layout. Since the actual cutting volume was small and there was almost no wear on the single-point diamond tooltip, the effect of residual rounding angle on the grating diffraction efficiency can be disregarded for the time being.

### 4.3. The Effect of the Workpiece Material on the Roughness of the Blaze Surface

**Figure 14:**
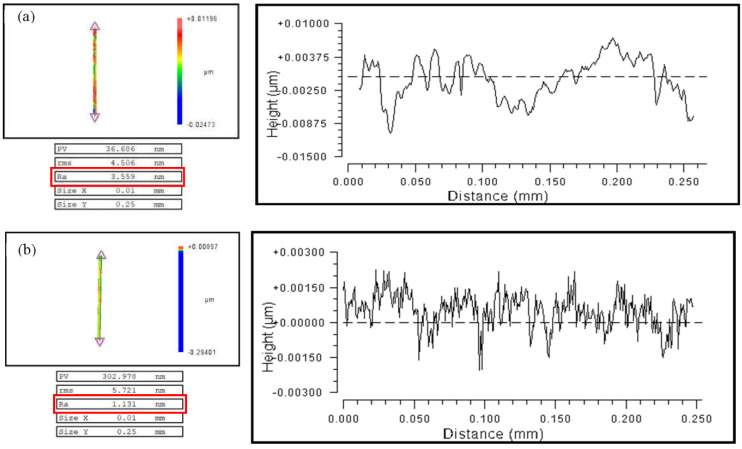
Roughness test of convex grating blaze surface of different workpiece materials along the equal-along-projection layout (**a**) RSA6061 (**b**) RSA6061+ chemically plated NiP.

The blaze surface roughness of a single blazed grating was investigated using the MetroPro software’s Mask Data tool. After filtering the substrate’s spherical waveform, a rectangular area of 0.01 mm × 0.25 mm was chosen. The average blaze surface roughness Ra of the convex grating along the equal-along-projection layout was up to 3.645 nm on the RSA6061 workpiece surface, while the average blaze surface roughness Ra of the blazed grating with the equal-along-arc layout was up to 3.689 nm, with both grating layouts having almost no effect on the blaze surface roughness. Furthermore, according to the above analysis, the convex blazed grating was machined on the surface of the RSA6061+ chemically plated NiP workpiece using the same cutting parameters and equal-along-projection layout. The RSA6061+ chemically plated NiP workpiece’s typical convex grating blazing surface roughness curve is illustrated in Figure 14b. Its surface roughness Ra reached 1.131 nm. The measured roughness of the 15 blaze surfaces could be arithmetically averaged to 1.523 nm, which was half of the roughness value of the RSA6061 workpiece blaze surface, but both fulfilled the criteria of the surface roughness of optical components being less than 10 nm.

### 4.4. The Effect of Workpiece Material on the Height of the Poisson Burr

**Figure 15:**
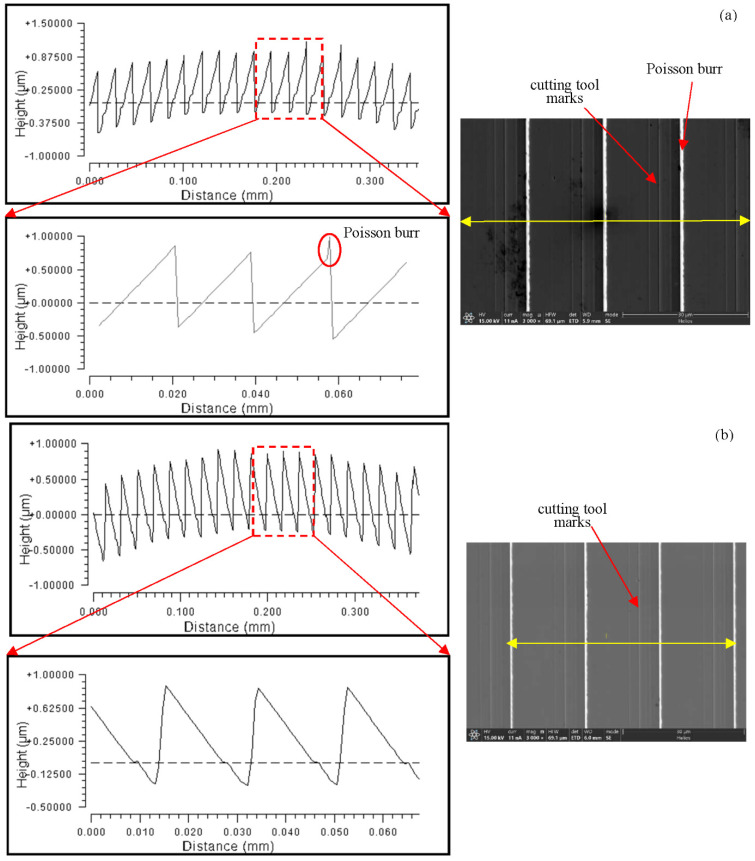
Comparison of Poisson burrs on the top of the grating for different workpiece materials along the equal-along-projection layout. (**a**) RSA6061 (**b**) RSA6061+ chemically plated NiP.

The influence of residual rounding on grating diffraction efficiency may be ignored for the time being because the actual cutting volume was minimal, and the single-point diamond tooltip showed essentially little wear before and after the experiment. Poisson burrs were found on the top of some of the blazed grating units on the RSA6061 workpiece with a height measurement of about 0.3 μm, which was the critical value, due to the plastic flow of the material, as shown in Figure 15, while no obvious Poisson burrs were found on the RSA6061+ chemically plated NiP workpiece. This is because RSA6061+ chemically plated NiP had a higher hardness value than RSA6061 and had a lower flow stress, indicating that the RSA6061+ chemically plated NiP material may have effectively reduced the development of Poisson burr on the top of the grating. When comparing the SEM pictures of the convex gratings of the two materials, it could be seen that the RSA6061 workpiece’s convex grating surface created evident cutting tool marks, whereas the RSA6061+ chemically plated NiP workpiece’s convex grating surface did not.

## 5. Conclusions

According to the spectral range and diffraction requirements of the imaging spectrometer, the structure size of the convex spherical blazed grating unit and the machining accuracy were determined. The ultra-precision four-axis machining system was used to design the tool geometry angle and optimize the cutting parameters according to the machining scheme. The surface roughness Ra was less than 5 nm, and the shape accuracy met the diffraction efficiency requirement. The minimum resolution according to the Z-axis was 200 nm, so the limit of the groove frequency that could be processed was 5000 line/mm. It was possible to apply this processing method to fabricate the blazed gratings used for visible wavelengths.

(1) Simulation results of the effect of different residual rounded corners and Poisson burr sizes on the diffraction efficiency of the actual profile of the convex spherical blazed grating showed that the diffraction efficiency of the grating gradually decreased in the spectral range as the residual rounded corners and Poisson burr sizes increased. When the Poisson burr height is less than 0.5 μm, the effect of Poisson burrs on the grating diffraction efficiency can be neglected.
(2) The use of low spindle speed in the ultra-precision machining system can effectively reduce the actual cutting speed, which is conducive to the entry of cutting fluid into the first deformation zone and chip removal. In addition, it can avoid large fluctuations in cutting force caused by changes in cutting temperature and can improve the machining accuracy of microstructures.
(3) Under the same cutting parameters, the grating with an equal-along-projection layout and equal-along-arc can maintain the dimensional accuracy of the grating period. However, in terms of grating height dimensional accuracy, the equal-along-arc layout is superior to equal-along-projection. In terms of blaze angle dimensional accuracy, equal-along-projection is superior to equal-along-arc.
(4) Both grating layouts have the same roughness of blaze surface, and the dimensional accuracy of the grating period is close, but the diffraction efficiency is superior that of the equal-along-arc layout due to the higher accuracy of the blaze angle of the equal-along-projection layout.
(5) The RSA6061+ chemically plated NiP material is superior for diamond turning of convex blazed gratings, because it has fewer Poisson burrs on the top of the grating and the blaze surface roughness value is lowered to Ra1.523 nm.

## Figures and Tables

**Table 1:** Specifications of convex spherical grating.

Parameter	Content
Wavelength	0.95–2.5 μm (SWIR)
Surface profile	Convex spherical
Surface shape	Circular
Substrate radius of curvature (mm)	41.104
Clear aperture/Diameter (mm)	14
Groove frequency (line/mm)	53.97
Substrate material	Optical grade aluminum
Coating material	Gold
Incidence angle (°)	−25.962
Diffraction order used	−1
Max diffraction efficiency	>82%

**Table 2:** Tool geometry parameters.

No.	Type	Rake Angle (°)	Clearance Angle (°)	Included Angle (°)	Tool Nose Radius (μm)	Cutting Edge Radius (μm)
1	R-shaped diamond tool	0	10	60	200	0.1
2	V-shaped diamond tool	0	15	110	0.1	0.1

**Table 3:** Tool and workpiece material properties.

Material	Density ρ/g·cm^−3^	Young’s Modulus E/GPa	Tensile/Compressive Strength б_b/_MPa	Yield Strength σ_0.2_/MPa	Thermal Conductivity /W·m^−1^·K^−1^	Poisson’s Ratio	Hardness
RSA6061	2.7	70	330	300	160	0.33	110 HB
RSA6061+ chemically plated NiP	7.75	50~70	700	\	4.19	0.3	485 HB
Single crystalline diamond	3.5	960	2000	N.A.	2000	0.2	8000 HB

**Table 4:** Dimensions and roughness measurements of convex spherical blazed grating elements.

Grating Layouts	Random Sampling Area	Grating Period/*d*		Grating Height/*h*		Blaze Angle/*θ_b_*		Blaze Surface Roughness (nm)
		Average Value (μm)	Deviation	Average Value (μm)	Deviation	Average Value (deg)	Deviation	
Equal-along-projection	1	18.3	−1.23%	1.23750	2.03%	3.89952	0.81%	3.89
	2	18.3	−1.23%	1.28228	5.72%	3.86385	−0.11%	3.611
	3	18.7	0.92%	1.27487	5.11%	3.92659	1.51%	3.443
	Average value	18.43	−0.53%	1.26488	4.29%	3.89665	0.74%	3.645
Equal-along-arc	1	18.3	−1.23%	1.20314	−0.80%	3.62372	−6.32%	3.817
	2	18.3	−1.23%	1.16814	−3.69%	3.69477	−4.48%	3.285
	3	18.4	−0.70%	1.16703	−3.78%	3.76656	−2.63%	3.966
	Average value	18.33	−1.07%	1.17944	−2.76%	3.69502	−4.48%	3.689
